# Supplementary material for: Seminoma and Embryonal Carcinoma Footprints Identified by Analysis of Integrated Genome-Wide Epigenetic and Expression Profiles of Germ Cell Cancer Cell Lines
Source: PLoS One. 2014 Jun 2;9(6):e98330. doi: 10.1371/journal.pone.0098330 (PMC4041891; doi:10.1371/journal.pone.0098330)
Supplement: File S1 — ZIP file containing DMRforPairs output for significant regions. Please start from the html files. (ZIP) [file pone.0098330.s008.zip › figures/41095.pdf]

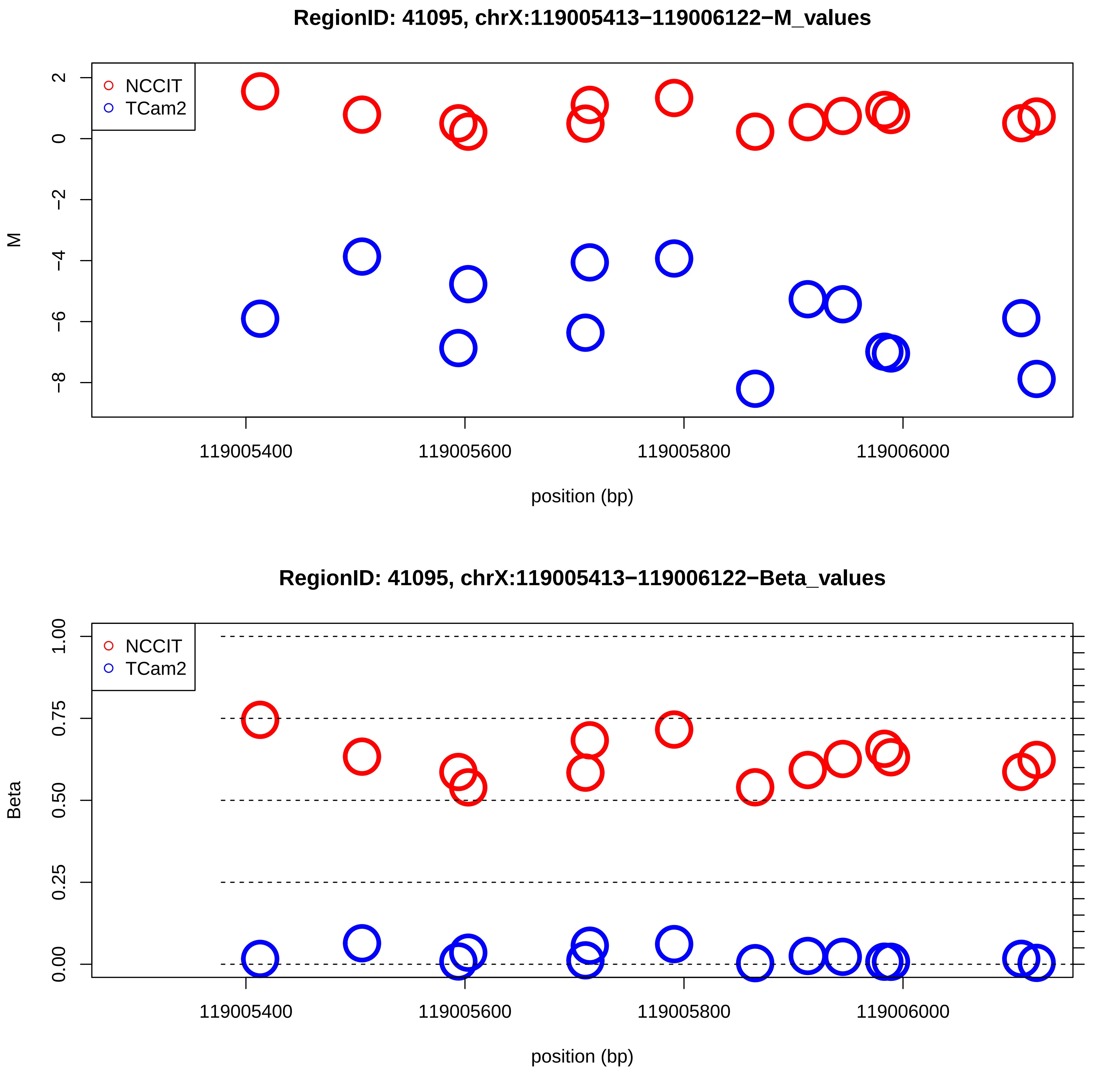

RegionID: 41095, chrX:119005413−119006122−M_values
NCCIT
TCam2
M
2
0
−2
−4
−6
−8
119005400
119005600
119005800
119006000
position (bp)
RegionID: 41095, chrX:119005413−119006122−Beta_values
NCCIT
TCam2
Beta
1.00
0.75
0.50
0.25
0.00
119005400
119005600
119005800
119006000
position (bp)

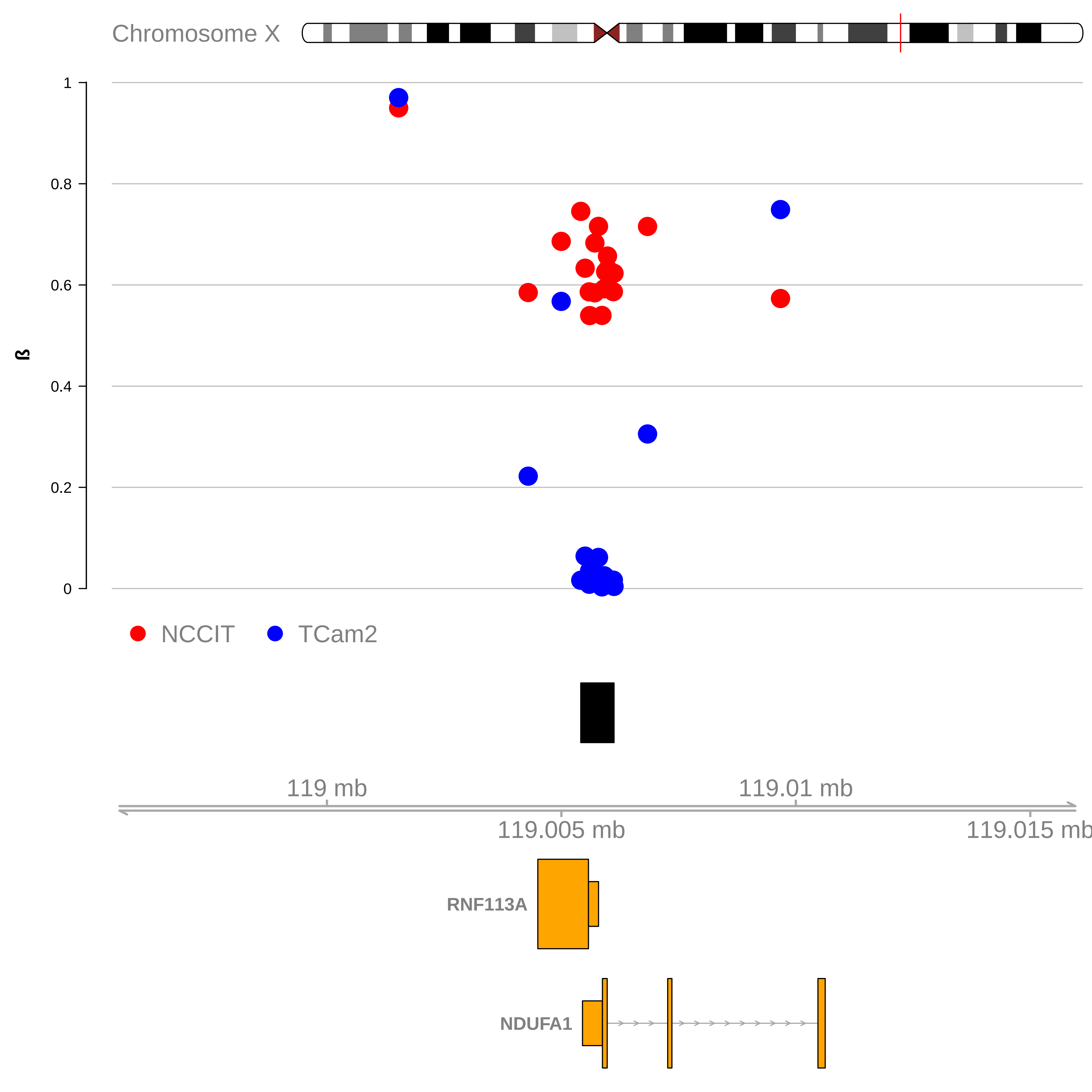

Chromosome X
1
0.8
0.6
0.4
0.2
0
ß
NCCIT
TCam2
119 mb
119.01 mb
119.005 mb
119.015 mb
RNF113A
NDUFA1
